# Hemophilic Pseudotumor of the Maxilla Secondary to Endodontic Treatment: Case Report and Systematic Review

**DOI:** 10.3390/dj13110491

**Published:** 2025-10-23

**Authors:** Jose Rodolfo Quiroz-Gomez, Carlos Manuel Roa-Encarnación, Ana Graciela Puebla-Mora, Antonio Hernández-Morales, Miguel Padilla-Rosas, Mario Nava-Villalba

**Affiliations:** 1 Master’s Program in Oral Pathology and Medicine, Department of Integral Dental Clinics, University Center of Health Sciences, Universidad de Guadalajara, Guadalajara 44340, Mexico; jose.quiroz9886@alumnos.udg.mx (J.R.Q.-G.); miguel.prosas@academicos.udg.mx (M.P.-R.); 2 High Specialty Medical Unit—Pediatrics, National Medical Center of the West, Instituto Mexicano del Seguro Social, Guadalajara 44340, Mexico; roec_@hotmail.com; 3 Pathology Research and Diagnostic Center, Department of Microbiology and Pathology, University Center of Health Sciences, Universidad de Guadalajara, Guadalajara 44340, Mexico; graciela.puebla@academicos.udg.mx; 4 Faculty of Dentistry, Autonomous University of the Mexico State, Toluca de Lerdo 50130, Mexico; ahernandezmo@uaemex.mx

**Keywords:** hemophilic pseudotumor, endodontic complication, oral pathology

## Abstract

Hemophilic pseudotumor (HP) is a rare but severe complication of hemophilia, characterized by progressive bleeding in the muscles, joints, and bone tissue, which can lead to lytic lesions. Its prevalence is approximately 1–2% among patients with hemophilia. This report presents a male patient with mild hemophilia A who developed an intraosseous lesion in the posterior region of the right maxilla, with a prior history of endodontic treatment in the area. Surgical excision was performed and, following clinicopathological correlation, the lesion was diagnosed as HP. **Background/Objectives:** This review aims to identify previously reported cases of HP located in the maxilla. **Methods:** The study protocol followed the Preferred Reporting Items for Systematic Review and Meta-Analyses (PRISMA) guidelines. The databases PubMed, Scopus, and ScienceDirect were searched, and Google Scholar was used to identify gray literature. The Joanna Briggs Institute (JBI) tool was employed to assess the risk of bias and the quality of the included reports and case series. **Results:** A total of 1487 publications were identified using specific keywords. After removing duplicates and non-relevant titles/abstracts, 42 full-text articles were reviewed. Of these, 10 met the inclusion criteria: 7 case reports and 3 case series, comprising 13 cases of HP in the maxilla, including the case presented here. Although rare in the maxillofacial region, when it does occur, it is more commonly seen in the mandible and is often linked to prior trauma. In this case, endodontic treatment may have triggered lesion development. **Conclusions:** This report highlights that, although uncommon, HP can manifest with involvement of the maxilla, and that specific dental interventions may represent potential triggering events.

## 1. Introduction

Hemophilia-associated pseudotumors are severe complications of this congenital condition, with a prevalence of 1 to 2%. They were first described by Starker in 1918 and are associated with high morbidity and mortality. They most commonly develop in the femur, tibia, pelvic bones, and iliac bones, which are constantly at risk of trauma. However, in rare cases, they may occur in the craniofacial region, specifically involving the maxillary bone. To date, 12 cases have been reported in this region [1,2].

Hemophilia patients are relatively rare in the population, affecting one in every 10,000 people. Hemophilia is classified into three main types according to the deficient coagulation factor: hemophilia A (factor VIII deficiency), hemophilia B (factor IX deficiency), and hemophilia C (factor XI deficiency). While 95% of cases are either type A or B, which are X-linked recessive disorders, type C is transmitted in an autosomal recessive pattern and can occur in both sexes. In addition to these congenital forms, hemophilia can be acquired, most often due to the development of autoantibodies against coagulation factors. Acquired hemophilia has been reported in association with autoimmune diseases, malignancies, pregnancy, and skin conditions [3]. It is also classified according to its severity, divided into mild, moderate, and severe [4]. Hemophilic pseudotumor (HP) is the result of confined hemorrhages in muscles, joints, and bone tissue, which, as they grow, cause increasing osmotic pressure, leading to inflammation and a lytic lesion due to ischemia. It is generally painless unless nerve areas are affected. Although the term “hemophilic pseudotumor” suggests occurrence exclusively in patients with hemophilia, it can also develop in individuals in other bleeding diatheses or coagulopathies like von Willebrand disease, since the pathogenesis of the lesion is very similar across these conditions [5,6].

HP’s clinical manifestations are not well defined and depend on the affected area, influenced by the anatomical region where it is located and the adjacent structures it could compromise. It can range from a slowly developing cystic lesion to a bone fracture with joint dysfunction. In the jaws, it is usually described as a rapidly growing swelling due to constant trauma in the area, which can be mistaken for an aggressive tumor [7,8]. It is essential for dentists to be aware of the patient’s medical history, particularly the presence of bleeding disorders such as hemophilia. Depending on the type of dental procedure—especially those involving extensive manipulation of soft or hard tissues—serious complications may arise. For this reason, several clinical protocols have been proposed and are currently available to guide the safe and effective management of patients with hemophilia in dental settings [9].

## 2. Materials and Methods

**Figure 1:**
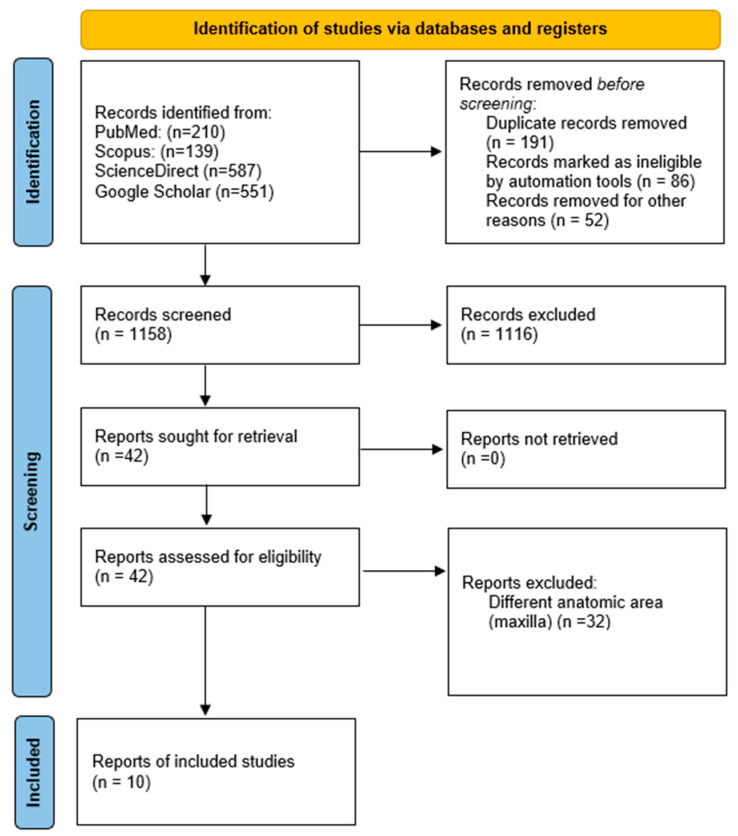
Flowchart summarizing the article selection process (*n*—number of studies).

For this systematic review, the Preferred Reporting Items for Systematic Review and Meta-Analyses (PRISMA) recommendations were adhered to (Figure 1), as well as the CARE guidelines for case reports. The following focus questions were formulated: What was the occurrence by age, association with etiological factors, and prior diagnosis of hemophilia in patients with hemophilic pseudotumor in the maxilla? The objective of this review is to identify previously described cases of this entity in the maxilla [10,11].

### 2.1. Eligibility Criteria

The inclusion criteria were clinical case reports, case series, and systematic reviews on HP and similar nomenclatures such as hemophilic cyst or hemophilic tumor; in this case, localized in the maxilla or affecting the upper jawbones. Documents with sufficient clinical and histological information to confirm the diagnosis and location were included. There were no restrictions regarding the language of the report or the publication period.

### 2.2. Exclusion Criteria

Exclusion criteria included scientific documents that did not meet the inclusion criteria (conference abstracts, book chapters, letters to the editor, etc.), as well as cases that did not involve localization or involvement of the upper maxilla, or those that lacked sufficient information to confirm the diagnosis and location.

### 2.3. Sources of Information and Search

For the electronic literature review, the databases PubMed, Scopus, and ScienceDirect were used. For gray literature, Google Scholar was employed, and data were collected until September 2024. For PubMed, the following search strategy was used (Pseudotumor, Hemophilia [MeSh]) AND (Hemophilic pseudotumor) AND (Hemophilic cysts) AND (Hemophilic pseudo tumor) AND (Haemophilic pseudotumor) AND (Haemophilic cysts) AND (Heamophilic pseudo tumor) AND (Maxilla) OR (Gnathic) NOT (Mandibule) NOT (Animal). For ScienceDirect, the search strategy was: (Pseudotumor hemophilia) AND (Hemophilic pseudotumor) AND (Hemophilic cysts) AND (Hemophilic pseudo tumor) AND (Haemophilic pseudotumor) AND (Haemophilic cysts) AND (Haemophilic pseudo tumor) AND (Maxilla) OR (Gnathic) NOT (Mandibule) NOT (animal) NOT (Jaw). For Scopus, the search strategy was: TITLE-ABS-KEY (Pseudotumor AND Hemophilia) OR (Hemophilic AND Cysts) OR (Hemophilic AND Pseudo AND Tumor) OR (Haemophilic AND Cysts) OR (Haemophilic AND Pseudo AND Tumor) AND (Maxilla) OR (Gnathic) OR (Oral) AND NOT (Mandibule) AND NOT (Animal). In Google Scholar, the following search strategy was used: “Pseudotumor hemophilia” OR “Hemophilia tumor” OR “haemophilia-associated pseudotumour” OR “Hemophilic pseudo tumor” OR “hemophilic cysts” AND “Maxilla” AND “Gnathic” AND “Oral” (Appendix A).

### 2.4. Study Selection

For case selection, abstracts were fully read if they did not violate the inclusion criteria. If they met the criteria, the full article was reviewed to discern its relevance and select appropriate documents.

### 2.5. Data Collection and Summary Measures

For data collection, the following variables were analyzed and extracted: authors, type of publication, year of publication, sex, age, exact location, treatment, follow-up, and the type of hemophilia the patient had (Table 1). All these data were analyzed using descriptive statistics.

### 2.6. Quality Assessment of Individual Studies

The quality of the studies was independently evaluated following the Joanna Briggs Institute (JBI) guidelines, which consider criteria such as a complete clinical history and detailed demographic characteristics for evaluating the case. These criteria were assessed with the options “Yes”, “No”, “Not applicable”, and “Unclear”. The studies were classified according to their quality into three levels: high bias (when the study reached up to 49% of the scores), moderate bias (50–69%), and low bias (over 70%) (Table 2 and Table 3) [18].

## 3. Results

### 3.1. Case Report

**Figure 2:**
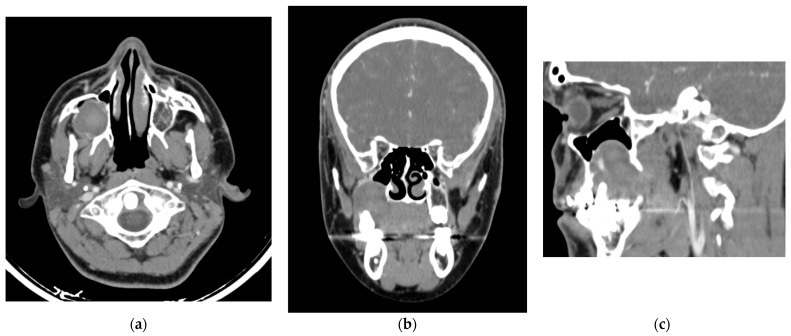
Contrast-enhanced computed tomography (**a**) Axial slice reveals an isodense area relative to soft tissues with hyperenhancing central regions, show prominent vascularization. (**b**) Coronal view revealing elevation of the right maxillary sinus associated with the maxillary first molar. (**c**) Sagittal view illustrating in greater detail the sinus elevation and regions of hyperenhancement.

**Figure 3:**
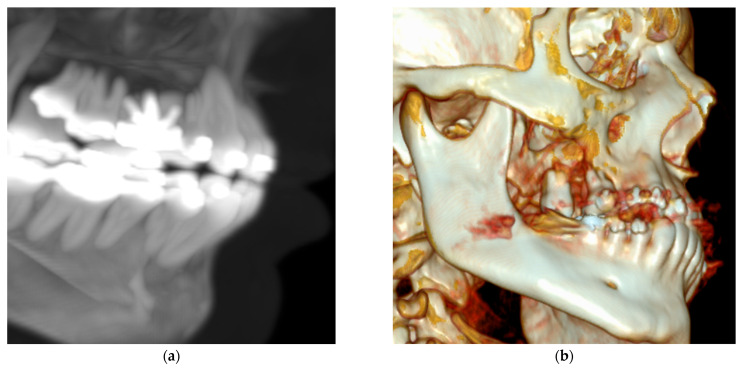
Contrast-enhanced computed tomography (**a**) Three-dimensional reconstruction with MIP mask showing the endodontic treatment performed on the upper first molar. (**b**) Three-dimensional reconstruction showing bone loss/erosion in the right maxilla, primarily affecting the molars.

**Figure 4:**
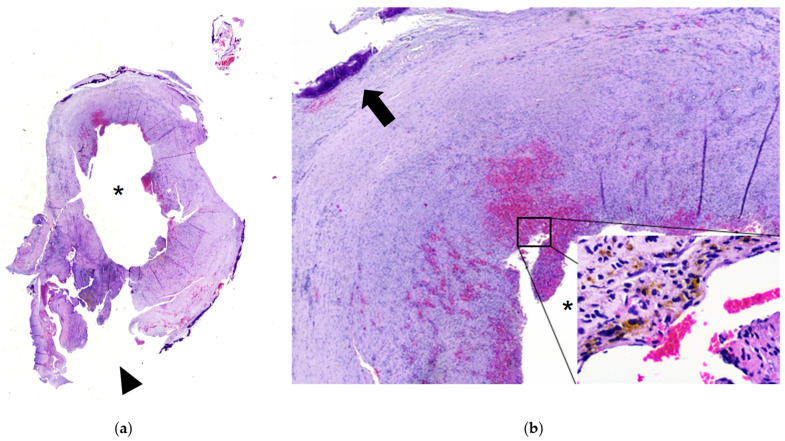
Whole slide image digitalization of histological tissue stained with hematoxylin and eosin (**a**) Panoramic magnification showing the pseudocystic conformation of the tissue with luminal surface (asterisk), cortical loss with muscle continuity (arrowhead); (**b**) Low magnification microscopic image showing a fibrous connective tissue capsule, with cortical thinning observed in the superior region (arrow), the luminal surface (asterisk), which is not covered by any epithelium and instead shows granulation tissue with tissue hemorrhage; inset, a high-magnification view highlights a hemosiderin deposit near the luminal area.

A 14-year-old male with mild hemophilia A, currently undergoing orthodontic treatment, presented to the Department of Oral and Maxillofacial Surgery (UMAE—Pediatrics, CMNO—IMSS) with facial asymmetry in the right zygomatic region due to an asymptomatic expansile mass. On intraoral examination, a swelling was observed in the right maxillary region, extending from the second premolar to the second molar, producing elevation of the vestibular sulcus, the lesion was associated with dental mobility and occasional bleeding. Panoramic radiography revealed a well-demarcated radiolucent intraosseous lesion in the posterior maxilla, with elevation of the maxillary sinus floor. The patient’s parents reported a history of endodontic treatment in the same region approximately four months earlier. Computed tomography demonstrated a well-defined lesion in the right maxilla with centrally hyperenhancing areas, suggestive of increased vascularity (Figure 2 and Figure 3). Preoperative management consisted of replacement therapy with recombinant factor VIII, which was administered to achieve and maintain normal hemostatic levels prior to the surgical intervention. Once adequate correction of coagulation parameters was confirmed, the surgical procedure was carried out under strict hematologic supervision. Conservative curettage and careful debridement of the pseudotumor were performed to minimize surgical trauma and extraction of the involved maxillary first molar. Intraoperative hemostasis was optimized using local measures, including absorbable hemostatic agents and primary closure with resorbable sutures. Postoperatively, replacement therapy was maintained to sustain factor levels above 50% during the healing phase, and antifibrinolytic agents were prescribed as adjuvant therapy. Gross examination revealed a pseudocystic lesion surrounded by a fibrous connective tissue capsule. Histopathologic analysis showed a lesion wall composed of numerous blood vessels, hemosiderin deposits, and granulation tissue, with no evidence of epithelial lining. The lesion was well circumscribed, except in one area showing cortical bone loss (Figure 4). Due to spindle cell morphology and hypercellularity, immunohistochemical staining for alpha smooth muscle actin (SMA) was performed to differentiate from entities such as angioleiomyoma and myofibroma. A focal area of cortical discontinuity raised suspicion for nodular fasciitis; however, β-catenin immunostaining was negative. Cluster of Differentiation 34 (CD34) immunostaining highlighted reactive blood vessels, while Kiel 67 (Ki-67) showed peripheral labeling, supporting an expansile growth pattern (Appendix B). Clinicopathologic correlation supported a final diagnosis of HP.

### 3.2. Systematic Review

In our initial search across the four consulted databases, we found a total of 1487 scientific documents: Scopus (*n* = 139), PubMed (*n* = 210), ScienceDirect (*n* = 587), and Google Scholar (*n* = 551). Duplicates and documents whose titles and abstracts were not applicable to our objective were removed, leaving a total of 42 articles that were reviewed in full text. Of these, only 10 documents met our inclusion criteria: 7 clinical case reports and 3 case series, reporting 13 cases of hemophilic pseudotumor in the maxilla, including the one presented in this document.

The oldest reported case was from 1995 [6], and the most recent in 2022 [17], excluding the clinical case reported here. Twelve cases were reported in male patients, and one in a female patient [14]. Ages ranged from 1 year to 75 years, with the highest prevalence in the second decade of life, and an average age of 18.9 years. Eight cases were treated with surgery followed by replacement therapy, three with replacement therapy only, and two with radiotherapy.

Nine patients had hemophilia type A, two had hemophilia type B, one had von Willebrand disease, and one did not have hemophilia. Seven cases reported moderate hemophilia, two cases reported mild hemophilia, two cases reported severe hemophilia, one case was unspecified, and one case did not have hemophilia. Six cases were reported in China, two in Brazil, and the remaining were single cases reported in countries such as Korea, New Zealand, the United States, Japan, and Mexico (present case). According to JBI criteria, one case was classified as low bias^5^, and 11 with high bias [2,3,6,16,17].

## 4. Discussion

### 4.1. Gender

Most HP predominantly occurs in males due to the X-linked recessive inheritance pattern of hemophilia, which means that males, having only one X chromosome, are more susceptible to developing the condition. However, rare cases in females have been documented. One such case involved a female patient with HP in the maxillary region, suggesting that while exceedingly uncommon, HP can manifest in females, particularly in those with von Willebrand disease or symptomatic carriers of hemophilia [14].

### 4.2. Age

HP is most frequently diagnosed in children and young adults, primarily in the first and second decades of life. This trend aligns with the natural history of hemophilia, where complications tend to arise early due to repeated bleeding episodes. However, Stevenson et al. [12] reported an exceptional case in a patient over 70 years old, highlighting the possibility of late-onset HP in rare circumstances. This deviation from the usual age distribution suggests that factors such as trauma, residual clotting factor activity, or additional comorbidities may contribute to late presentations [2].

### 4.3. Type of Hemophilia

Most of the patients had hemophilia type A, while two had hemophilia type B. Additionally, one case involved a patient with von Willebrand disease [14], and interestingly, one case of pseudotumor was reported in a patient without hemophilia [12]. This is consistent with case series of HP in various locations, where hemophilia A is the most reported [1]. Most patients had moderate to severe hemophilia, making them more prone to complications. Our case involved a patient with mild hemophilia, which makes them less likely to develop such events, but like our report, Yong et al. [3] also described a case with mild hemophilia. One female patient with HP was diagnosed with von Willebrand disease after the pseudotumor was her first clinical manifestation [14]. No cases of HP in the maxilla were reported as the first clinical manifestation of hemophilia, unlike cases in other locations, such as the mandible [7].

### 4.4. Location

A classification of HP has been proposed based on the affected tissue type: Type I occurs in soft tissue (14.3%), Type II in the subperiosteal region, and Type III within bone (85.7%) [15]. Hemophilic pseudotumors are commonly found in long bones like the femur or tibia, and in small bones such as those in the hand, likely because these are more prone to trauma. In the head and neck region, most reported cases involve the mandible, involved in 75% of the cases, while few affects other bones, such as the maxilla, as this area is less exposed to frequent trauma compared to the mandible [19].

### 4.5. Etiology

HP formation typically requires two primary factors: a bleeding disorder (such as hemophilia) and a triggering traumatic event. The specific type of trauma responsible for HP development remains unclear, though acute trauma is often implicated. However, some case series, such as those by Feng Xue et al. and Yang et al. [5,13] report that only a minority of maxillofacial HP cases had a history of trauma, suggesting that spontaneous bleeding episodes might also contribute [5]. Our review identified three cases, including our own, with a documented trauma history [6,15]. Additionally, the functional occlusal pressure in the posterior dental region during mastication ranges from 6 to 20 MPa [20]. Notably, our case involved chronic trauma related to an endodontic complication, a previously unreported risk factor. Endodontic issues, such as iatrogenic perforation, canal blockage, instrument separation, or untreated anatomy, can lead to prolonged inflammation and potential bleeding, creating a favorable environment for HP development. The clear spatial and temporal relationship between the endodontic intervention and HP occurrence in our case underscores the need for increased awareness of dental trauma as a potential etiological factor [21]. In our case, there is a clear relationship between the endodontic treatment and the development of the HP, given the lesion’s location and the temporal proximity.

### 4.6. Clinical Manifestations

The clinical manifestations of HP vary depending on the anatomical location and the type of tissue affected. In intraosseous lesions, they typically present as cystic, expansive, and asymptomatic structures, but they can cause fractures or dysfunction in the area. When affecting soft tissues, it behaves as an expanding hematoma [8]. In the maxilla, four cases reported swelling and bleeding as clinical manifestations [14,15,16,17], while three cases reported spontaneous gingival bleeding [2,3,5], consistent with HP cases in the mandible. Other manifestations in the maxillary region include epistaxis and proptosis [12,13], likely due to the proximity of the maxilla to other important anatomical structures. Dental mobility was present in our case as an early sign of changes; similarly, Siqueira et al. reported dental mobility near the lesion [2].

### 4.7. Histology

Hemophilic pseudotumor is histologically characterized by a pseudocystic structure with an empty or hemorrhagic lumen, a thick fibrous wall, and recurrent or abundant intracapsular hemorrhage, often with hemosiderin deposits. Few reports have described the immunohistochemical profile of this entity. In the case report by Cai et al. [16] markers such as Glucose Transporter 1 (GLUT-1) and Cluster of Differentiation 31 (CD31) were employed. In contrast, our case involved a more comprehensive immunohistochemical panel, aimed at excluding other entities considered in the differential diagnosis. A focal area of cortical discontinuity raised suspicion for nodular fasciitis, prompting the use of β-catenin immunostaining, which yielded negative results. Consequently, SMA immunostaining was performed to help exclude diagnoses such as myofibroma and angioleiomyoma, which frequently share overlapping histological features. This was particularly relevant given the consideration of intraosseous myofibroma, a lesion typically observed during the second decade of life. SMA staining showed focal positivity. Additionally, CD34 and Ki-67 immunostaining were performed to further characterize the lesion. CD34 expression was limited to reactive blood vessels, and Ki-67 showed peripheral labeling, consistent with an expansile growth pattern.

In routine cases, the histopathological features of HP, in conjunction with clinical correlation—particularly a known history of hemophilia—are usually sufficient to establish the diagnosis. However, in the present case, the history of hemophilia was not communicated when the specimen was initially submitted for histopathological evaluation. The rarity of HP in the maxillary bones further complicated the diagnostic process, necessitating consideration of alternative differential diagnoses as described. This case underscores the importance of thoroughly reviewing the patient’s clinical history and ensuring effective communication between medical, dental, and pathology teams involved in the care of patients with complex bleeding disorders [2,5,16].

### 4.8. Treatment and Prognosis

All cases, except for the patient without hemophilia, were treated with factor replacement therapy. However, only three cases received this treatment exclusively [2,3,6]. In eight cases, factor replacement was combined with surgery [2,5,14,15,16,17], and in two cases, radiotherapy was used, one exclusively in a patient without hemophilia, due to multiple complications that precluded surgical management, the patient received a concentrated dose of 30 Gy radiotherapy, which led to the resolution of complications and an uneventful 18-month follow-up. Another case in combination of radiotherapy with factor replacement therapy, also with positive outcomes. Radiotherapy is recommended in cases where surgery is not advisable, due to the patient’s health or age, but it should be administered in low doses [3,9,12]. The most recent case reported in the maxilla was treated with surgery, factor replacement therapy, and the use of humanized monoclonal antibodies, specifically emicizumab, which was successful in the long-term treatment of this complication [17]. Although there is no standardized treatment protocol HP particularly in cases involving the maxilla and mandible, surgical management remains the cornerstone of therapy for this entity. Nevertheless, increasing emphasis has recently been placed on the critical role of perioperative replacement therapy—both preoperative and postoperative—which is essential to ensure adequate hemostasis and to minimize the risk of complications. This therapeutic approach highlights the need for an interdisciplinary management strategy, involving close collaboration between oral and maxillofacial surgeons, hematologists, and other specialists, to achieve favorable outcomes [22].

## 5. Conclusions

In conclusion, this systematic review identified a total of 13 cases of hemophilic pseudotumor in the maxillary region, reported by 10 authors. Among these is a newly reported case in a patient with mild hemophilia. This case also includes an immunohistochemical panel that aids in the differential diagnosis, allowing the hemophilic pseudotumor to be distinguished from other histological similar entities. The reviewed cases were categorized according to their clinical features, histology, and etiology, among other criteria, providing a deeper understanding of how these factors influence the presentation of this condition and highlighting its potential association with dental procedures.

## Figures and Tables

**Table 1:** Overview of all included studies.

Study (Author(s), Year)	Country	Type of Study	Gender	Age (Years)	Location	Treatment	Follow-Up (Months/Years)	Type of Hemophilia	Intensity of Hemophilia
Machado de Sousa 1995 [6]	Brazil	Case series	Male	11	Left maxilla second premolar to molar	Replacement therapy	A month	Type A	Severe
Stevenson 2002 [12]	New Zealand	Case report	Male	75	Left maxillary sinus	Radiotherapy	18 months	Without hemophilia	NR
Siqueira 2008 [2]	Brazil	Case report	Male	12	Left posterior maxillary region	Surgery and replacement therapy	9 months	Type A	Moderate
Xue 2011 [5]	China	Case series	Male	24	Left maxillary sinus	Replacement therapy	NR	Type A	Moderate
Yang 2012 [13]	China	Case series	Male	1	Right posterior maxillary region	Replacement therapy	36 months	Type B	Moderate
Yang 2012 [13]	China	Case series	Male	2	Left maxillary sinus	Surgery and replacement therapy	6 months	Type A	Moderate
Yang 2012 [13]	China	Case series	Male	2	Left posterior maxillary region	Surgery and replacement therapy	7 years	Type A	Moderate
Argyris 2016 [14]	United States	Case report	Female	11	Right posterior marginal border of maxilla	Surgery and replacement therapy	NR	von Willebrand disease	NA
Kwon 2016 [15]	Korea	Case report	Male	53	Hard palate and floor of right maxillary sinus	Surgery and replacement therapy	3 months	Type B	Moderate
Cai 2020 [16]	China	Case report	Male	11	Left maxilla	Surgery and replacement therapy	5 years	Type A	NR
Yong 2020 [3]	China	Case report	Male	11	Right maxilla	Radiotherapy and replacement therapy	10 years	Type A	Mild
Kawahara 2022 [17]	Japan	Case report	Male	14	Left maxilla	Surgery, replacement therapy and antibody treatment	19 months	Type A	Severe
Present case 2025	México	Case report	Male	14	Left maxilla	Surgery and replacement therapy	12 months	Type A	Mild

NR: Not Referred; NA: Not Applicable.

**Table 2:** Results of the quality assessment for cases series following the Joanna Briggs Institute (JBI) guidelines checklist.

Author(s)	Q 1	Q 2	Q 3	Q 4	Q 5	Q 6	Q 7	Q 8	Q 9	Q 10	Overall Score and Quality
Yang [13]	Y	Y	N	Y	Y	Y	Y	Y	Y	Y	90% (High)
Machado de Sousa [6]	Y	Y	U	N	Y	Y	Y	Y	Y	Y	80% (High)
Xue [5]	N	N	N	N	N	Y	Y	Y	Y	Y	50% (Low)

Y: Yes; N: No; U: Unclear; Q: Question.

**Table 3:** Results of the quality assessment for cases reports following the JBI.

Author(s)	Q 1	Q 2	Q 3	Q 4	Q 5	Q 6	Q 7	Q 8	Overall Score and Quality
Stevenson [12]	Y	Y	Y	Y	Y	Y	Y	Y	100 (High)
Siqueira [2]	Y	Y	Y	Y	Y	U	Y	Y	87.5 (High)
Argyris [14]	Y	Y	Y	Y	Y	Y	Y	Y	100 (High)
Kwon [15]	Y	Y	Y	Y	Y	U	Y	Y	87.5 (High)
Cai [16]	Y	U	Y	Y	Y	Y	Y	Y	87.5 (High)
Yong [3]	Y	Y	U	Y	Y	U	Y	Y	75 (High)
Kawahara [17]	Y	Y	Y	Y	Y	Y	Y	Y	100 (High)
Our case	Y	Y	Y	Y	Y	Y	Y	Y	100 (High)

Y: Yes; N: No; U: Unclear; Q: Question.

**Figure A1:**
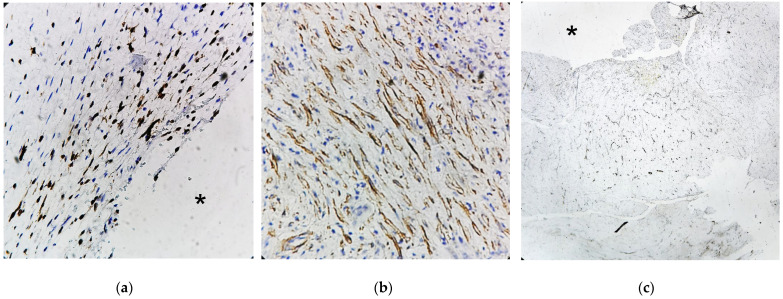
Microscopic images of the immunostaining panel (**a**) Strong label demonstrating nuclear immunoreactivity for the Ki-67 marker in peripheral cells of the lesion adjacent to the trabeculae (asterisk), indicating an osteolytic and expansive/proliferative process (Magnification 10×). (**b**) Strong label showing spindle-shaped cells with cytoplasmic immunoreactivity for smooth muscle actin, consistent with myofibroblasts in a focal pattern (Magnification 10×). (**c**) Low-magnification image showing immunoreactivity for the CD34 marker in blood vessels concentrated toward the pseudoluminal surface of the lesion (asterisk) (Magnification 4×).

## Data Availability

The original contributions presented in this study are included in the article. Further inquiries can be directed to the corresponding author.

## Abbreviations

The following abbreviations are used in this manuscript:

HP	Hemophilic pseudotumor
PRISMA	Preferred Reporting Items for Systematic Review and Meta-Analyses
JBI	The Joanna Briggs Institute
SMA	Alpha smooth muscle actin
CD34	Cluster of Differentiation 34
Ki-67	Kiel 67
GLUT-1	Glucose Transporter 1
CD31	Cluster of Differentiation 31

## Appendix A. Electronic Literature Review Search Strategy

**Databases Database Link Search Date**	**Search Strategies**	**Number of Articles Found**
PudMed	(Pseudotumor, Hemophilia [MeSh]) AND (Hemophilic pseudotumor) AND (Hemophilic cysts) AND (Hemophilic pseudo tumor) AND (Haemophilic pseudotumor) AND (Haemophilic cysts) AND (Heamophilic pseudo tumor) AND (Maxilla) OR (Gnathic) NOT (Mandibule) NOT (Animal).	210
ScienceDirect	(Pseudotumor hemophilia) AND (Hemophilic pseudotumor) AND (Hemophilic cysts) AND (Hemophilic pseudo tumor) AND (Haemophilic pseudotumor) AND (Haemophilic cysts) AND (Heamophilic pseudo tumor) AND (Maxilla) OR (Gnathic) NOT (Mandibule) NOT (animal) NOT (Jaw)	587
SCOPUS	TITLE-ABS-KEY (Pseudotumor AND Hemophilia) OR (Hemophilic AND Cysts) OR (Hemophilic AND Pseudo AND Tumor) OR (Haemophilic AND Cysts) OR (Haemophilic AND Pseudo AND Tumor) AND (Maxilla) OR (Gnathic) OR (Oral) AND NOT (Mandibule) AND NOT (Animal)	139
Google Scholar	“Pseudotumor hemofilia” or “Tumor hemofilia” or “haemophilia-associated pseudotumour” or “Hemophilic pseudo tumor” or “haemophilic cysts” and “Maxilla” and “Gnatico” and “Oral”.	551
